# Early Detection of Physical Function Decline in Older Adults by Identifying Gut-Microbiome Based Biomarkers

**DOI:** 10.1093/geroni/igaf122.2540

**Published:** 2025-12-31

**Authors:** Sridevi Nair, Yi Lin, Shalini Jain, Rohit Shukla, Vivek Kumar, Robert Mankowski, Hongdao Meng, Hariom Yadav

**Affiliations:** University of South Florida, Tampa, Florida, United States; The University of Alabama at Birmingham, Birmingham, Alabama, United States; University of South Florida, Tampa, Florida, United States; University of South Florida, Tampa, Florida, United States; University of South Florida, Tampa, Florida, United States; The University of Alabama at Birmingham, Birmingham, Alabama, United States; University of South Florida, Tampa, Florida, United States; University of South Florida, Tampa, Florida, United States

## Abstract

Decline in physical function in older adults leads to frailty, falls, fractures, reduced quality of life, and increased healthcare utilization, resulting in a significant financial burden on individuals, families, and healthcare systems. Currently, there are no effective tools to predict the risk of physical function decline in older adults. Emerging evidence suggests the gut microbiome plays a key role in maintaining muscle, joint, and overall physical function; however, its prognostic potential and underlying mechanisms remain poorly understood. In this study, we utilized data from the Florida state-wide Microbiome in Aging Gut and Brain (MiaGB) consortium to explore the relationship between the gut microbiome and physical function. We identified gut bacterial taxa and metabolic pathways associated with physical performance. Using Receiver Operating Characteristic (ROC) curve analysis, we demonstrated that specific microbial signatures accurately distinguish between individuals with low and high physical function. Furthermore, we analyzed correlations between physical function and microbial metabolic pathways, identifying operons linked to these pathways. Notably, we found that chondroitin metabolism pathways were significantly impaired in older adults with lower physical function compared to those with normal function. These findings highlight gut microbiome signatures as potential biomarkers for predicting physical function decline and suggest that impaired chondroitin metabolism may be a key microbial mechanism contributing to musculoskeletal deterioration in aging. This study provides novel insights into the microbiome’s role in age-related physical decline and offers opportunities for early prognosis and development of microbiome-based interventions to prevent or mitigate these debilitating conditions in older adults.

